# Pathogen-Specific Epitopes as Epidemiological Tools for Defining the Magnitude of *Mycobacterium leprae* Transmission in Areas Endemic for Leprosy

**DOI:** 10.1371/journal.pntd.0001616

**Published:** 2012-04-24

**Authors:** Marcia V. S. B. Martins, Marjorie M. da S. Guimarães, John S. Spencer, Mariana A. V. B. Hacker, Luciana S. Costa, Fernanda M. Carvalho, Annemieke Geluk, Jolien J. van der Ploeg-van Schip, Maria A. A. Pontes, Heitor S. Gonçalves, Janvier P. de Morais, Tereza J. P. G. Bandeira, Maria C. V. Pessolani, Patrick J. Brennan, Geraldo M. B. Pereira

**Affiliations:** 1 Laboratory of Cellular Microbiology, Oswaldo Cruz Institute, FIOCRUZ, Rio de Janeiro, Brazil; 2 Department of Microbiology, Immunology and Pathology, Colorado State University, Fort Collins, Colorado, United States of America; 3 Leprosy Laboratory, Oswaldo Cruz Institute, FIOCRUZ, Rio de Janeiro, Brazil; 4 Laboratory of Medical Informatics, School of Medical Sciences, State University of Rio de Janeiro, Rio de Janeiro, Brazil; 5 Department of Infectious Diseases, Leiden University Medical Center, Leiden, The Netherlands; 6 Dona Libânia Reference Center, Fortaleza, Brazil; 7 Leprosy Control Program/SER V/Fortaleza, Fortaleza, Brazil; 8 LabPasteur Diagnostic Medicine/Diagnostics of America (DASA), Fortaleza, Brazil; 9 Laboratory of Immunopathology, School of Medical Sciences, State University of Rio de Janeiro, Rio de Janeiro, Brazil; Institut Pasteur, France

## Abstract

During recent years, comparative genomic analysis has allowed the identification of *Mycobacterium leprae*-specific genes with potential application for the diagnosis of leprosy. In a previous study, 58 synthetic peptides derived from these sequences were tested for their ability to induce production of IFN-γ in PBMC from endemic controls (EC) with unknown exposure to *M. leprae*, household contacts of leprosy patients and patients, indicating the potential of these synthetic peptides for the diagnosis of sub- or preclinical forms of leprosy. In the present study, the patterns of IFN-γ release of the individuals exposed or non-exposed to *M. leprae* were compared using an Artificial Neural Network algorithm, and the most promising *M. leprae* peptides for the identification of exposed people were selected. This subset of *M. leprae*-specific peptides allowed the differentiation of groups of individuals from sites hyperendemic for leprosy versus those from areas with lower level detection rates. A progressive reduction in the IFN-γ levels in response to the peptides was seen when contacts of multibacillary (MB) patients were compared to other less exposed groups, suggesting a down modulation of IFN-γ production with an increase in bacillary load or exposure to *M. leprae*. The data generated indicate that an IFN-γ assay based on these peptides applied individually or as a pool can be used as a new tool for predicting the magnitude of *M. leprae* transmission in a given population.

## Author Summary

Despite the efforts to treat registered leprosy patients, the number of new cases reported globally remains stable and high (about 200,000/year). As the treatment of multibacillary leprosy patients, the major recognized source for new infections, did not allow the expected reduction in new leprosy cases, additional sources must be considered. Following exposure to *M. leprae* infection, the evolution to active disease is estimated to take from 2 to 10 years, and it is conceivable that some of these asymptomatic individuals could be a yet unrecognized source of infection. Previously, the use of computational tools allowed us to select *M. leprae*-specific genes or gene regions, and derive *M. leprae*-specific synthetic peptides from the *M. leprae* genome. *Ex vivo* stimulation of the blood leukocytes with a subset of these peptides induced IFN-γ production that allowed the differentiation of individuals exposed to *M. leprae* from unexposed ones. Individuals with no known history of exposure to *M. leprae*, but living in an area with high frequency of leprosy cases had high-level positive responses to the peptides. This last observation raised the possibility of using this test as a tool for evaluating the level of transmission of *M. leprae* infection in areas of interest.

## Introduction

Leprosy is a chronic infectious disease caused by the obligate intracellular pathogen *Mycobacterium leprae*. Multidrug therapy (MDT), a combination of antibiotics very effective in curing this mycobacterial infection, was introduced by WHO in the early eighties. With the success of MDT, in 1991 the World Health Assembly set a target of eliminating leprosy as a public health problem by the year 2000. Elimination was defined as reaching prevalence levels of <10 case per 100,000 individuals. The elimination program has been successful in delivering MDT worldwide, decreasing globally the number of registered cases. More than 13 million cases were detected and treated with MDT from 1982 to 2002 [1]. However, the disease is still considered a public health problem in several countries (www.who.int/lep). Particularly in Brazil, the new-case detection rate remains high and stable at approximately 40,000 new cases annually (http://portal.saude.gov.br) indicating that transmission has not been adequately interrupted in Brazil by treating leprosy patients with MDT alone. Therefore new strategies and approaches need to be developed in order to definitively eradicate leprosy as a public health problem. This conclusion is supported by recent mathematical modeling of leprosy indicators suggesting that leprosy is slowly declining but the rate of decline is uncertain, requiring sustained leprosy control efforts [2].

Leprosy manifests itself as a spectrum of clinical forms, but which for treatment purposes has been simply divided into multibacillary (MB) and paucibacillary (PB) leprosy. In Brazil, the detection rate among children under 15 years of age is stable and considered high (approximately 8% of the new cases detected yearly) indicating that despite a high level of MDT coverage by the National Leprosy Control Program (PNCH), active transmission persists (http://portal.saude.gov.br). Leprosy transmission is still poorly understood. The major sources of the bacteria are considered to be the multibacillary patients, carrying a high bacterial load in their skin and being able to shed large numbers of bacteria from their nasal passages; 10^7^ viable *M. leprae* per day on average [3]. Two major pitfalls have contributed to our poor understanding of leprosy transmission: the long incubation period of the disease estimated to be between 2 and 10 years; and the absence of a test that could specifically measure exposure to *M. leprae*, since the magnitude of the *M. leprae*-infected population is much higher than that of individuals with actual leprosy. A serological test based on the detection of antibodies specific for the phenolic glycolipid-I (PGL-I) antigen, a unique molecule of the *M. leprae* cell wall, is positive in most MB patients but not in PB individuals, showing a positive correlation with the bacterial load [4]. New tools that could detect infected/exposed individuals are desperately needed to measure the level and dynamics of leprosy transmission. Moreover, most of the infected individuals will never develop the disease [5]. Thus, a test that could distinguish between exposed/infected individuals and those evolving to active disease, should allow for early diagnosis and subsequent prevention of disabilities as well as stoppage of the transmission chain.

Mycobacteria are intracellular pathogens and as such elicit in the host a specific cell-mediated immune response that controls the infection. CD4^+^ T helper 1 (Th1) lymphocytes play a central role in this response, producing IFN-γ that will activate the microbicidal mechanisms of macrophages leading to the killing of intracellular microorganisms (for a review see [6]). The protective role of IFN-γ has been emphasized in several reports that describe the deleterious effects on mycobacterial infections of mutations/deletions in the IFN-γ gene and its receptor [7]. Thus, assessment of T cell functions provides a good alternative approach for the diagnosis of mycobacterial infections.

In most endemic countries for leprosy, such as Brazil, tuberculosis is also endemic with 71,641 new cases detected in 2009 (http://portal.saude.gov.br). Moreover, in Brazil, BCG vaccination is prescribed. Thus, the development of an immunologic test that could specifically detect *M. leprae*-infected individuals has to take these facts into account. In this context, a new scenario was introduced by the genomic era, with the knowledge of whole chromosome sequences of mycobacterial species, particularly *M. leprae*, *M. tuberculosis* and *M. bovis* BCG, providing unique opportunities to identify *M. leprae* specific antigens. We have used comparative genome analysis to identify *M. leprae*-specific genes, and tested both recombinant proteins and synthetic peptides derived from a subset of these proteins to test for immunological reactivity [8]–[13]. In a previous study performed with individuals living in Rio de Janeiro, a panel of 58 peptides (15 mers and 9 mers) was tested for induction of IFN-γ responses in PBMCs of leprosy patients, healthy household contacts (HHC) of leprosy patients, TB patients, and endemic and non-endemic healthy controls [13]. Encouraging results were generated indicating that synthetic peptides induce specific responses in individuals exposed to *M. leprae* and could potentially be developed into a rapid test for the detection of *M. leprae* infection. In the present study, the 17 peptides with the best performance were selected and evaluated in individuals with different histories of exposure to *M. leprae* living in another endemic region of Brazil and in areas that are non-endemic for leprosy. The data generated indicate that an IFN-γ assay based on these peptides applied individually or as a pool can be used as a tool for predicting the magnitude of M. *leprae* transmission level in a given population.

## Materials and Methods

### Ethics statement

The tests and procedures described in this work were approved by the Oswaldo Cruz Foundation and D. Libania Ethics Committees. All subjects provided informed written consent.

### Study population

For the analysis of IFN-γ levels induced by *M. leprae*-derived peptides, a total of 127 volunteer subjects living in the city of Fortaleza, Ceará State, Brazil were enrolled. Untreated paucibacillary (PB) (three tuberculoid (TT) and 18 borderline tuberculoid patients [BT]), and multibacillary (MB) (eight lepromatous [LL] and thirteen borderline lepromatous patients [BL]) leprosy patients, household contacts of multibacillary patients (HCMB, n = 37), and household contacts of paucibacillary patients (HCPB, n = 27) were recruited from the Dona Libânia Reference Center, Fortaleza, Ceará, Brazil. Healthy individuals with no history of exposure to leprosy and/or tuberculosis were recruited from Bom Jardim (endemic controls high burden, EC_high_, n = 20) and Meireles (endemic controls medium burden, EC_low_, n = 18), Fortaleza districts with, respectively, hyperendemic (162 cases per 100 000 inhabitants) and medium (9 cases per 100 000 inhabitants) annual new case detection rates for the disease. Twenty one healthy blood donors recruited from the Blood Bank of the Fundação Estadual de Pesquisa e Produção em Saúde, Porto Alegre, Rio Grande do Sul State, Brazil (non-endemic controls, Brazil; NEC_Brazil_), and with no history of exposure to leprosy or tuberculosis were also included. Rio Grande do Sul was the first Brazilian State to achieve the WHO leprosy elimination goal in 2001 (a prevalence rate lower than 10 cases per 100,000 inhabitants). The 2009 new case detection rate in this State was 1.44/100,000. This State is, however, endemic for tuberculosis; with a detection rate of 46.14/100,000 in 2009 (http://portal.saude.gov.br). The baseline characteristics of each group of individuals included in the study are shown in **Table 1**. Two additional control groups, pulmonary tuberculosis patients who had received more than three months of treatment (tuberculosis, Netherlands; TB; n = 8) and healthy donors recruited at the Blood Bank Sanquin, Leiden, The Netherlands (non-endemic controls, Netherlands; NEC_Netherlands_; n = 8), all residents of the Netherlands, a non-endemic country for leprosy, were also included in the study.

**Table 1:** Baseline characteristics of Brazilian patients and healthy controls.

Group	N	M/F ratio	Age				BI mean
			Mean	25th	50th	75th	
NEC_Brazil_	21	13/8	32,71	7	12	2	-
EC_low_	18	6/12	34,89	6	11	1	-
EC_high_	20	9/11	33,35	5	15	-	-
HCPB	27	10/17	29,81	11	15	1	-
HCMB	37	11/26	37,35	10	18	9	-
PB	21	7/14	41,43	3	13	5	0
MB	21	12/9	42,8	4	10	7	2,72

### *M. leprae* whole cell sonicate

Irradiated armadillo-derived *M. leprae* whole cells were probe sonicated with a Sanyo sonicator to >95% breakage. This material was provided through the NIH/NIAID “Leprosy Research Support” Contract N01 AI-25469 from Colorado State University (these reagents are now available through the Biodefense and Emerging Infections Research Resources Repository listed at http://www.beiresources.org/TBVTRMResearch Materials/tabid/1431/Default.aspx).

### *In vitro* stimulation of PBMC with antigens

Blood was drawn by venipuncture, heparinized, and PBMC were isolated using Lymphoprep (Pharmacia Biotech, Uppsala, Sweden) by density gradient centrifugation, washed in PBS and resuspended in AIMV medium (Invitrogen, Grand Island, NY, USA) supplemented with 100 U/ml penicillin, 100 µg/ml streptomycin and 2 mM L-glutamine (Sigma Chemical, St. Louis, MO). PBMC from each individual were seeded at 2×10^5^ cells per well in 96-well round-bottomed plates in duplicate (BD Biosciences, San Jose, CA) and stimulated *in vitro* with armadillo-derived *M. leprae* whole cells (20 µg/ml), PPD (10 µg/ml) individual peptides (10 µg/ml), Pool 1[p52, p61, p68, p69] (0,1; 1,0 and 10 µg/ml), Pool 2 [p38, p51, p56, p59, p65, p67, p70, p71, p88, p91, p92] (0,1; 1,0 and 10 µg/ml)or staphylococcal enterotoxinB (SEB, 1 µg/ml) (Sigma). Cultures were incubated at 37°C in humidified 5% CO_2_ atmosphere. Supernatants were harvested at day-five of incubation and stored immediately at −70°C.

### IFN-γ ELISA

IFN-γ levels were determined in duplicate by ELISA (U-CyTech, Utrecht, The Netherlands). The cut-off value to define positive responses was set beforehand at 100 pg/ml. The assay sensitivity level was 40 pg/ml. Values for unstimulated cell cultures were typically <20 pg/ml [10].

### ELISA for detection of anti-PGL-I IgM

ELISA for detection of anti-PGL-I IgM was done as previously described [15]. The antigen used in ELISA was NT-P-BSA (synthetic native trisaccharide of PGL-I coupled to BSA through a 3-phenylpropanoyl) [14], and a cutoff value of 0.25, at an optical density at 450 nm (OD450) was set for positive responses [15].

### Statistical analysis

Artificial Neural Networks (ANN) modeling (Statistica Neural Networks 7, Statsoft, Tulsa, OK, USA) was used for the selection of peptides with the best performance in discriminating individuals with *M leprae* infection/disease based on its capacity to induce IFN-γ production in PBMC. The ANN model used for evaluating the peptides had 3 layers of neurons (Feedforward neural network). In the first or input layer, each node receives the IFN-γ level values in response to a given peptide. Each neuron of the first layer is connected to all the neurons of the second or hidden layer. The IFN-γ levels are multiplied by the weights or synaptic strengths before entering the neurons of the second layer. The second layer neurons integrate these processed values and if the resulting number is above an established threshold, this activates the delivery of a value to a synapse connecting the second layer neuron to the single neuron in the output layer. If the sum of the weighted numbers coming from the hidden layer is below the threshold of the output neuron, a “0” output is obtained, or if the value is above the threshold a “1” output is the result. The “0” was associated to one group (Ex. Non-exposed individuals) and the “1” to a second and expectedly different group (Ex. Individuals exposed/infected with *M. leprae*). The software trains the ANN by adjusting the synaptic values using the IFN-γ levels of individuals with known exposure/infection status. The ANN training is validated by analyzing a second group with no information made available to the ANN regarding infection status, and finally the ANN is used for testing with the complete groups to be differentiated. The performance of each peptide in discriminating different groups is evaluated by using the performance ratio (PR) of that peptide, a ratio between the mean square error (MSE) of the ANN with exclusion of the responses to the peptide (MSE_ex_) and the MSE of the ANN including the peptide (MSE_comp_; PR = MSE_ex_/MSE_comp_). Peptides with the PR values of less than “1” in a given ANN configuration were preferentially removed, and the performances of the ANN with and without those peptides compared in order to reach the best set of peptides for the discrimination of individuals exposed/infected with *M. leprae*, or with active disease.

Levels of IFN-γ responses to *M. leprae* peptides were compared among groups by Kruskal-Wallys test. Multiple tests were used to compute post-hoc comparisons for all the pairs of groups. A p value of 5% or less was considered significant. The analyses were performed with the STATISTICA software (Statsoft). Box plot graphs were done using the SPSS software (SPSS Inc, Clicago, IL, USA).

## Results

### Selection of *M. leprae*-specific peptides

**Figure 1:**
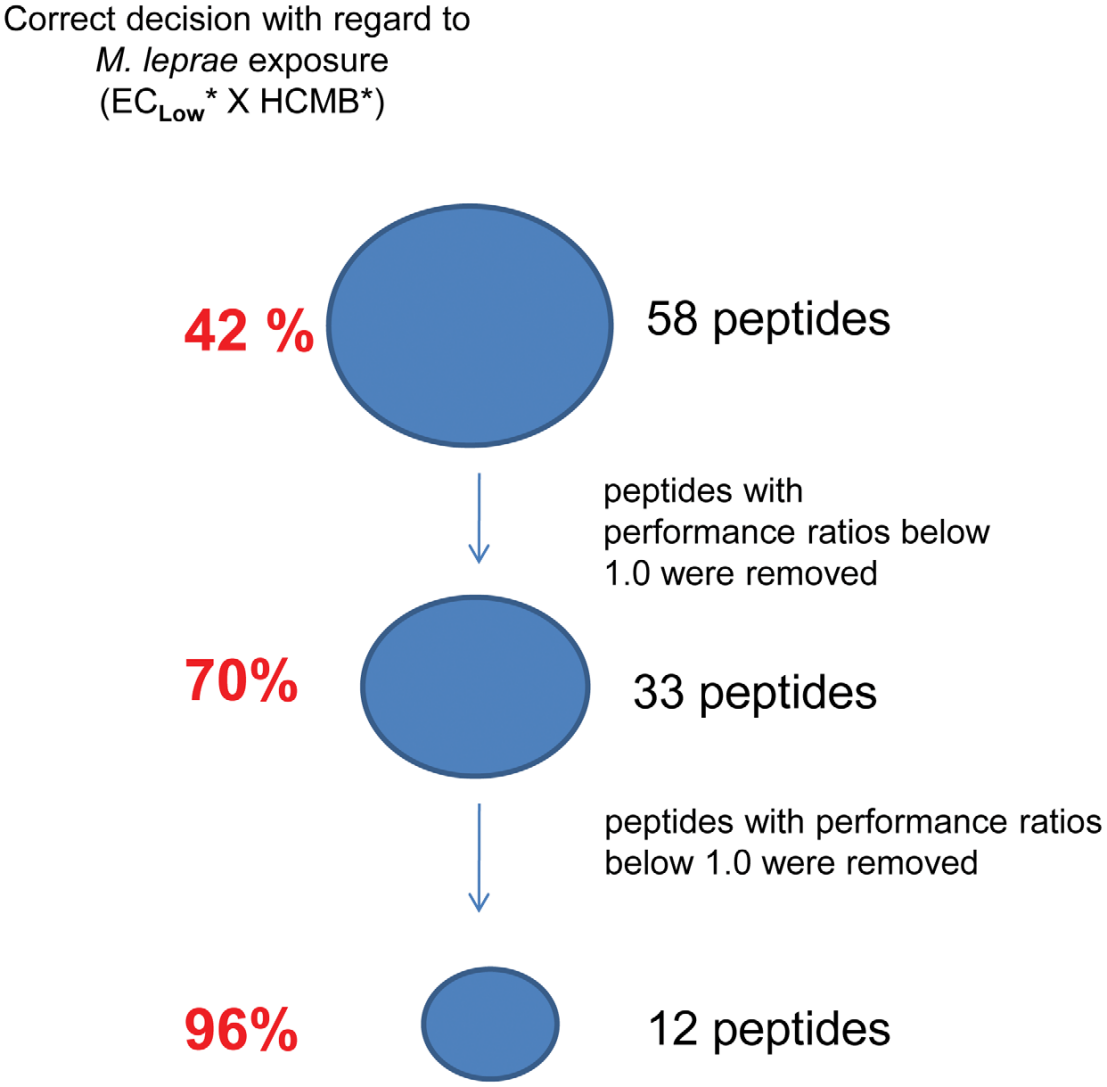
Selection of *M. leprae*-specific peptides. Fifty-eight *M. leprae*-specific peptides were previously tested for induction of IFN-γ release by PBMC from leprosy patients and contacts, endemic and non-endemic controls [13]. The IFN-γ levels induced by the peptides in non-exposed (EC_low_) and *M. leprae*-exposed individuals (HCMB) were used for selecting the best set of peptides allowing discrimination of the exposed group by applying an ANN algorithm. The final 12- peptide ANN made the right choice in 96% of the tests, identifying *M.leprae*-exposed or non-exposed individuals. *, individuals recruited from the city of Rio de Janeiro.

In order to identify people infected with *M. leprae* using an interferon-γ release assay, *M. leprae*-specific epitopes were required for *ex vivo* stimulation of the memory T cells of the individuals infected with this bacillus. The set of peptides had to be promiscuous, ideally binding to all the HLAs expressed in the population to be evaluated, to make sure that absence of IFN-γ detection would happen only in non-exposed people. The *in silico* analysis of genomes allowed the selection of *M. leprae*-specific genes and derived proteins. Previously we applied algorithms for identifying HLA-binding regions to these specific protein sequences. The chosen regions or epitopes were selected for binding to class I (9 mers) and class II (15 mers) HLA molecules [13]. Seventeen peptides were selected for this study from the original panel of 58 *M. leprae*-specific peptides. These peptides were previously tested for induction of IFN-γ release by PBMC from leprosy patients and contacts, endemic and non-endemic controls [13]. The IFN-γ levels induced by the peptides in non-exposed (EC_low_) and exposed individuals (HCMB) were used for selecting the best set of peptides allowing discrimination of the *M. leprae*-exposed from the non-exposed group, by applying an ANN algorithm (**Figure 1**). When the peptides with PRs below 0.96 were removed, a final step of selection by ANN ranked the 12 best peptides in terms of potential for discriminating *M leprae* infection/disease (**Table 2**). The final 12 peptides made the right choice in 96% of the tested individuals in defining the individual status regarding infection with *M. leprae* (**Figure 1**; **Table S1**). Five additional peptides from the original panel of 58 [13] but with PRs below 0.96 (**Table 2**) were included in the subsequent analysis.

**Table 2:** Ranking of selected *M. leprae*-specific peptides according to performance ratio in Artificial Neural Network assessment.

ML number	Peptide	Sequence	Location	PR
ML0008c	p38*	TRLLTVVVKQRSKAF	aa21–35	1.36
ML1419c	p69*	RLDGTTLEV	aa113–121	1.31
ML1553	p71*	LDIYTTLARDMAAIP	aa180–194	1.23
ML1057	p52*	QLLGQTADV	aa59–67	1.22
ML1419c	p68*	LLEEGVIVL	aa267–275	1.21
ML1057	p51*	AAALEQLLGQTADVA	aa54–68	1.18
ML1420	p70*	MQEYRGLTSHTPCCR	aa93–107	1.15
ML0638	p85	NYEVSPIFARWPRNR	aa49–63	1.09
ML1419c	p65*	EAVLLRLDGTTLEVE	aa108–122	1.05
ML0398c	p59	MLILGLLPAILPACG	aa15–29	1.04
ML0398c	p61	LILGLLPAI	aa16–24	1.02
ML0308	p56	FDEYRAMFALSAMDL	aa17–31	0.96
ML2452c	p92*	LQAYSNLFGRTSAMQ	aa28–42	0.91
ML2347	p91*	LATVQYDDRRRFTKE	aa301–315	0.89
ML1419c	p67*	SGRVTYLNPVGVKWM	aa51–65	0.87
ML1189c	p88*	DDIWRTLASAVITGN	aa55–69	0.86
ML1829	p73*	DAEWLKLTSLGLRPR	aa108–122	0.85

Peptides (n = 17) were ranked according to highest (>1.0, n = 11) to lowest (<1.0, n = 6) performance ratio (PR) according to the artificial neural network (ANN). These peptides were selected from the original set of 58 peptides analyzed in a previous work [13]. From this study, peptides that gave responses only in the leprosy patient group and household contacts, but not in the TB or EC (healthy endemic control) groups appear with an asterisk; bold indicates the peptide is from a hypothetical unknown protein.

### High-level of leprosy detection rate is associated with responsiveness to *M. leprae* specific peptides by individuals with no history of contact with leprosy patients

The study population consisted of individuals living in the city of Fortaleza, Ceará State, located in the Northeast region of Brazil. Fortaleza is a city with 2.5 million inhabitants (Brazilian Institute of Geography and Statistics, 2009), and divided administratively in 114 districts. Besides the leprosy patients and their household contacts recruited from the Dona Libânia Reference Center, two groups of healthy individuals with no history of previous contact with leprosy were enrolled in the study. These individuals had residential addresses in Meireles or Bom Jardim, two districts of Fortaleza with, respectively, medium (9 cases per 100,000 inhabitants) and hyperendemic leprosy new case detection rates (160 cases per 100,000 inhabitants).

**Figure 2:**
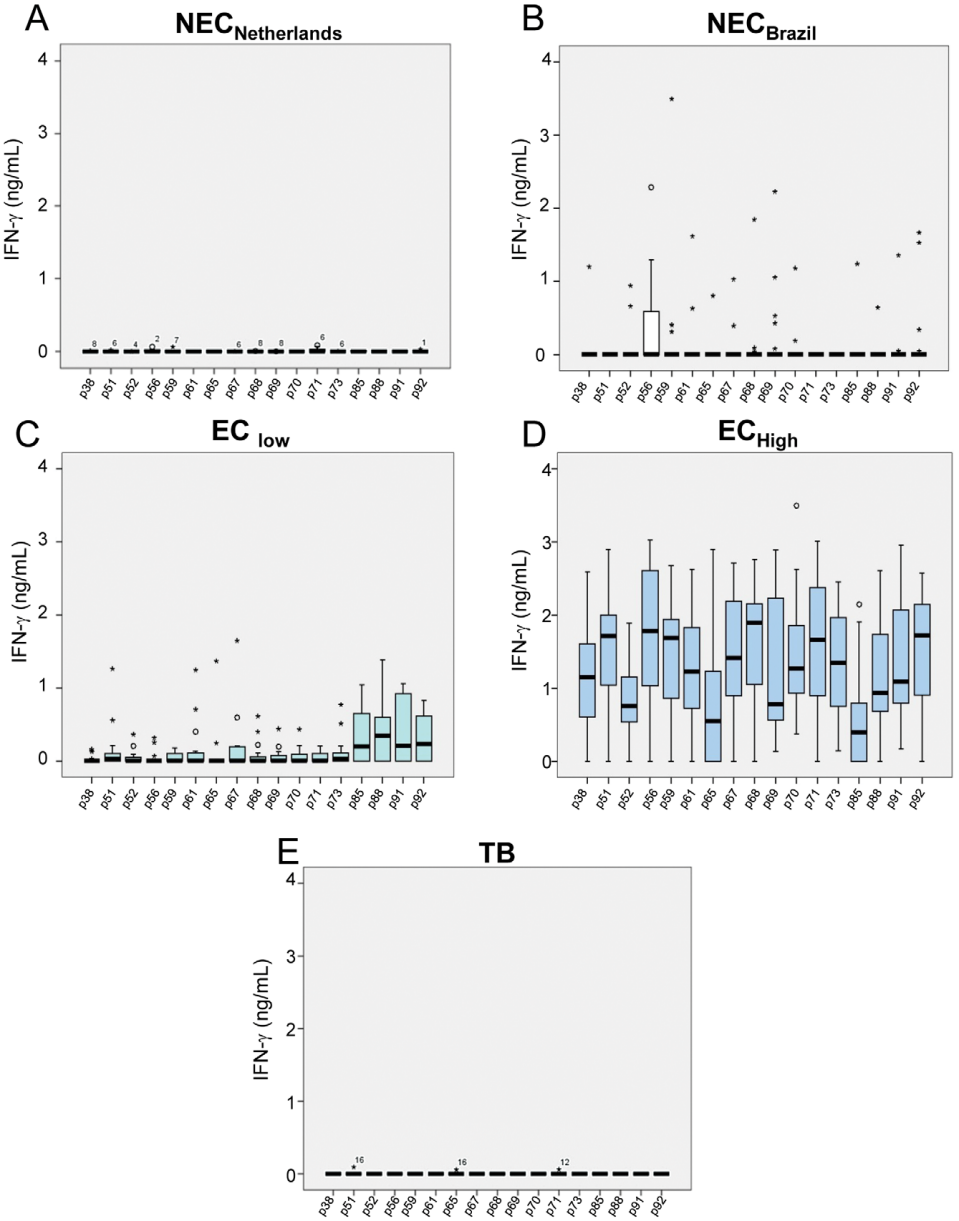
Leprosy detection rates and responsiveness to *M. leprae* specific peptides. Peripheral blood mononuclear cells (PBMCs) from individuals with different levels of exposure to *M. leprae* were stimulated with 17 *M.leprae*-specific peptides, and the concentration of IFN-γ measured in culture supernatants. The boxes include response rates of 75% of the sample, and the horizontal bars in bold identify the medians. Points outside the deviation correspond to outliers. ○, Values between 1.5 and 3 box lengths from the upper or lower edge of the box. *, Values more than 3 box lengths from the upper or lower edge of the box. **A**: A Dutch group of healthy non-endemic controls (NEC_Netherlands_); **B**: healthy controls from Porto Alegre, Brazil (NEC_Brazi_); **C**: healthy endemic controls from an area with medium annual new case detection rate for leprosy (Meireles, Fortaleza, CE, Brazil; EC_low_); **D**: another area with hyperendemic leprosy annual new case detection rate (Bom Jardim, Fortaleza, CE, Brazil; EC_high_); **E**: a Dutch group of tuberculosis patients (TB).

**Figure 3:**
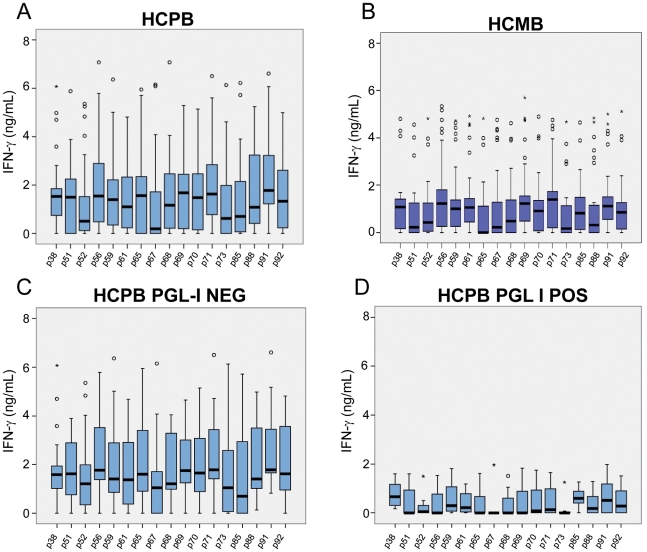
Responsiveness to *M. leprae*-specific peptides in household contacts of leprosy patients. The ex vivo stimulation of PBMC was done as described in Fig. 2 legend. **A**: Household contacts of paucibacillary (HCPB); and **B**: multibacillary leprosy patients (HCMB). The responses to the 17 *M. leprae*-specific peptides in PGL-I positive and negative HCPB are shown in separate plots (**C,D**). Detection of IgM anti-PGL1 in sera was performed using a specific ELISA.

**Figure 4:**
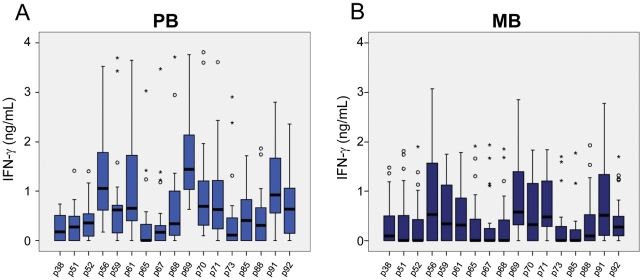
Responsiveness to *M. leprae*-specific peptides in PB and MB leprosy patients. The ex vivo stimulation of PBMC was done as described in Fig. 2 legend. **A**: Paucibacillary (PB); and **B**: multibacillary (MB) leprosy patients.

PBMC from leprosy patient groups (PB and MB), leprosy household contacts of PB (HCPB) and MB (HCMB) patients, healthy endemic controls from Meireles (EC_low_) and from Bom Jardim (EC_high_), healthy controls from Porto Alegre (NEC_Brazil_), Dutch tuberculosis patients (TB) and Dutch healthy, non-endemic controls (NEC_Netherlands_) were stimulated with peptides and control antigens, and IFN-γ was measured in culture supernatants on day 5 of incubation. The IFN-γ levels in the PBMC cultures of the different groups stimulated with the seventeen individual peptides are shown in the **Figures 2** **,** **3** **,** **4**. IFN-γ responses were below or, in a few cases, just above the detection limit, in all unstimulated cultures (medium alone) for all groups. All individuals responded well when their cells were cultured in the presence of the superantigen SEB (data not shown). **Figure 2 A–E** shows the responses of PBMC from groups of healthy individuals living in areas with different and increasing new case detection rates for leprosy (from zero to 162/100,000). Most of the Dutch individuals (TB and NEC_Netherlands_ groups) were responsive to PPD, but IFN-γ was below or, in a few cases, just above the detection limit in response to all *M. leprae* peptides, indicating absence of cross reactivity of the *M. leprae*-specific peptides in patients infected with *M. tuberculosis* or in BCG-vaccinated individuals (**Figure 2A, 2E**). The other control group enrolled in the study consisted of people living in Rio Grande do Sul, the first Brazilian State to achieve the WHO leprosy elimination goal (defined as a prevalence rate of lower than 10 cases per 100,000 inhabitants) in 2001. As shown in **Figure 2B**, most of the members of this group (NEC_Brazil_) produced undetectable levels of IFN-γ for all the peptides. A few outliers responded to the peptides, but the pattern of the group was clearly different from the observed in groups from endemic areas. However, evaluation of the IFN-γ responses of individuals living in Meireles (EC_low_) and Bom Jardim (EC_high_), districts of Fortaleza with medium and hyperendemic leprosy new case detection rates, 9 and 162 per 100,000 inhabitants, respectively, showed a marked contrast. Most members of the EC_low_ group displayed a reduced frequency of positive responses to the peptides. In contrast, members of the EC_high_ group showed good responses to all the peptides; the detection rate for leprosy is 18 times higher in the Bom Jardim district than in the Meireles district of Fortaleza. The groups of volunteers from these two sites had no history of known contact with leprosy patients; so, the levels of IFN-γ observed in the Bom Jardim individuals are consistent with the hypothesis that above a certain frequency of cases in the population, exposure to infection reaches the whole population, and history of contact with a leprosy patient is less relevant as an indicator of exposure to infection with *M. leprae*. **Figure 3 A–D** shows the IFN-γ levels observed in response to the peptides in groups of household contacts of leprosy patients (HCPB and HCMB). In general, lower levels of IFN-γ were observed in the HCMB group when compared to the HCPB. As expected, most of the PB patients responded to all the peptides (**Figure 4 A**). The same set of peptides, except for p85, also elicited IFN-γ production in MB leprosy patients although at a lower level (**Figure 4 B**).

**Figure 5:**
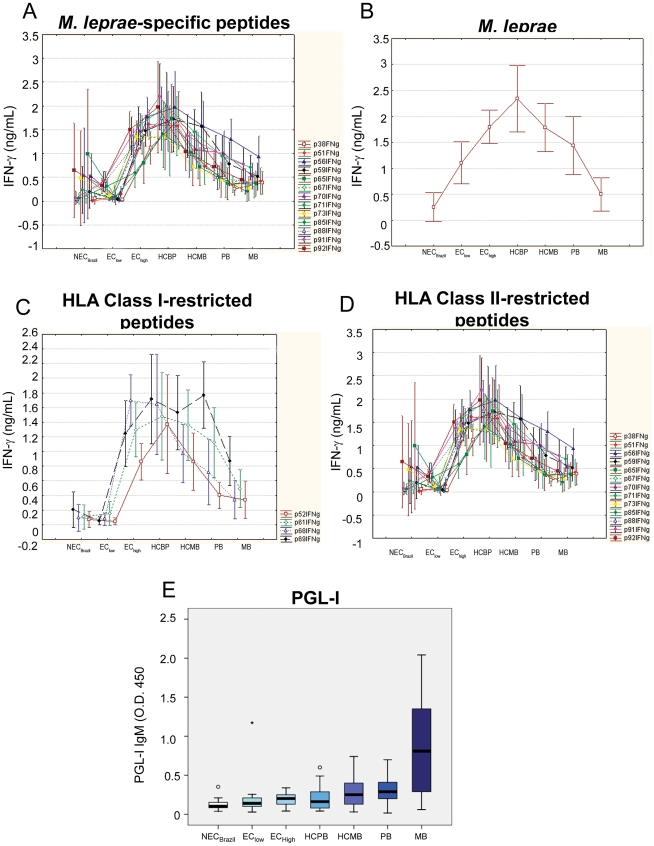
Level of exposure to *M. leprae* and response to the bacillus or *M. leprae*-specific peptides. Medians of the IFN-γ levels induced by *M.leprae*-specific peptides (**A, C, D**) or sonicated *M. leprae* (**B**) are shown for groups displayed in increasing order of exposure to *M. leprae* from left to right in the “x” axis. Detection of IgM anti-PGL1 in sera was performed using a specific ELISA (**E**).

The serum levels of anti-PGL-I IgM were measured in all of the Brazilian individuals enrolled in the study (**Figure 5E**). Assuming a cut off of 0.25, positivity to anti-PGL-I IgM was observed in 80% MB, 60% PB, 40% HCMB, 25% HCPB, 30% EC_high_, 10% EC_low_ and 10% NEC_Brazil_. An analysis of IFN-γ responses in positive vs. negative individuals showed significant differences in the HCPB group, with higher levels of IFN-γ produced by individuals who were anti-PGL-I negative (**Figure 3C, 3D**; differences were significant at a p<0.05 except for p59 and p85. Kruskal-Wallys test). No differences of the same kind were observed when PGL-I-positive and –negative HCMB were compared (**Figure S2**).

### Levels of IFN-γ in response to *M. leprae*-specific peptides are progressively reduced with increase in exposure to infection and in bacillary load

In order to link responses of the Brazilian groups of individuals with increasing exposure to *M. leprae* and increasing bacillary loads among the patients, the medians for the IFN-γ levels in response to the *M. leprae*-specific peptides were plotted simultaneously, and the resulting graphic shows an initial increase in the IFN-γ levels to all the peptides starting at baseline with the Brazilian non-endemic group (NEC_Brazil_) and the endemic controls in area of medium leprosy detection rate (EC_low_). IFN-γ levels peaked in the controls for hyperendemic areas (EC_high_) and contacts of paucibacillary leprosy patients (HCPB). The remaining groups show a progressive decline in IFN-γ levels that can be associated with continuous exposure to live *M. leprae* (HCMB) or increasing bacillary load for the two groups of patients (PB, MB) (**Figure 5A**). This observation was replicated when *ex vivo* levels of IFN-γ of the same groups to unfractionated *M. leprae* were evaluated (**Figure 5B**). This initial elevation and subsequent decline of IFN-γ levels in response to *M. leprae* was also observed when responses to only HLA class I- and HLA class II-restricted peptides were plotted (**Figure 5C, 5D**). This reduction in IFN-γ levels for *M. leprae* and *M. leprae*-derived peptides was not seen in the responses of the same groups to PPD and SEB (**Figure S1; Table S2**).

The down modulation of *M.leprae*-specific IFN-γ in the groups followed an inverse path when compared to the levels of PGL-I-specific IgM in the same groups (**Figure 5E**).

The evaluation of statistically significant differences between the groups can be summarized as follows: No difference was found when responses to the peptides of the NEC_Brazil_ and EC_low_ were compared. Nine to 16 peptides out of 17 induced markedly higher levels of response in exposed asymptomatic individuals (EC_high_, HCPB, HCMB) in comparison to the low-exposure or non-exposed individuals (NEC_Brazil_, EC_low_). In comparison to the exposed asymptomatic groups, the patients were responsive to a reduced number of peptides, but even the MB patients had responses that could be differentiated from the NECBrazil and/or EC low to 4 peptides (p56, p69, p71, p91). Another aspect that called the attention was the large number of peptides with reduced response in the MB patients in comparison to the exposed asymptomatic individuals (Kruskal-Wallys test, **Table S2**).

### Increase in the threshold for inducing IFN-γ production in response to pools of *M. leprae*-specific peptides is observed with increase in exposure to *M. leprae* or active disease

**Figure 6:**
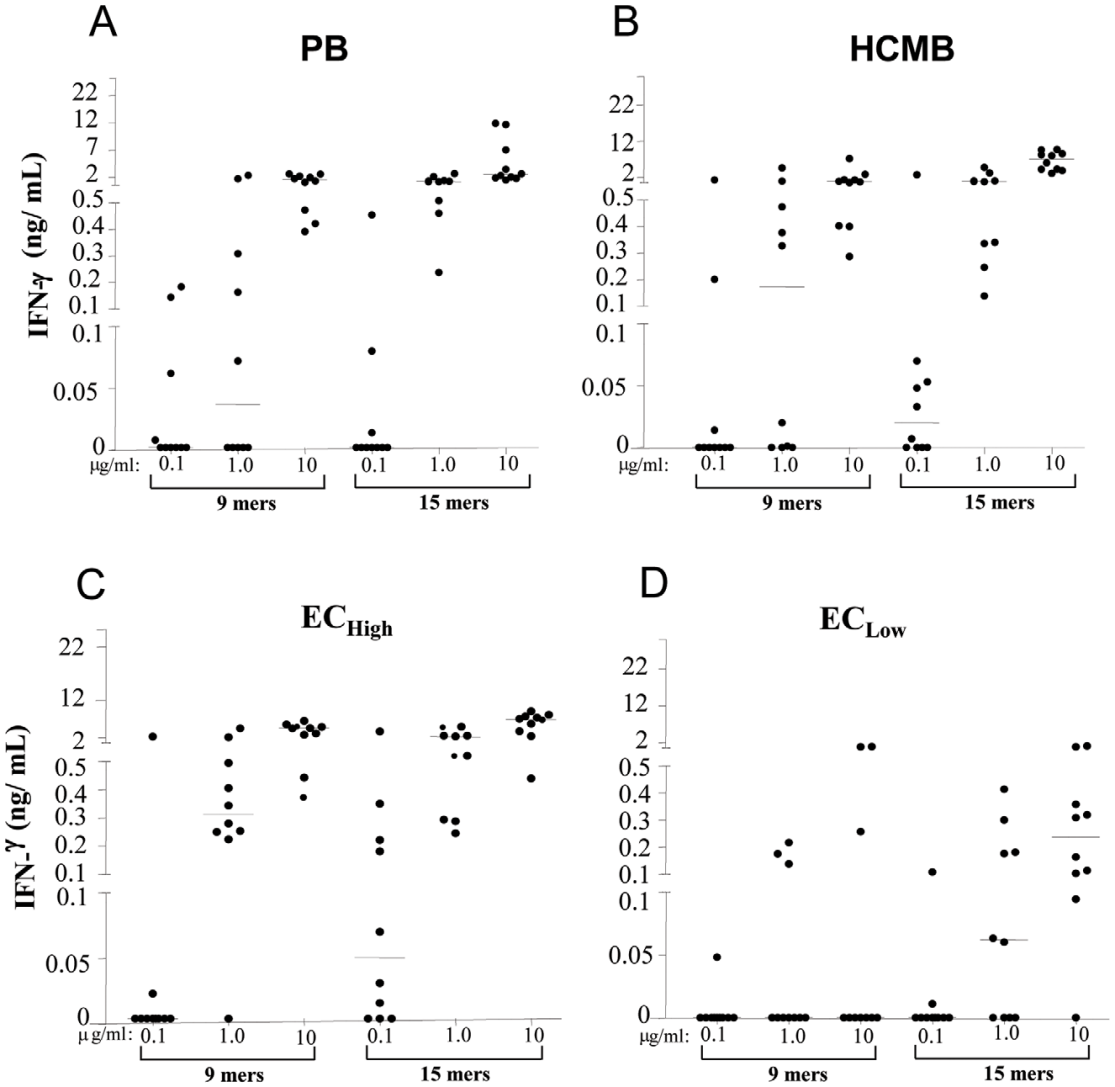
IFN-γ production in response to pools of *M. leprae*-specific peptides. PBMCs from PB (**A**), HCMB (**B**), EC_high_ (**C**) and EC_low_ (**D**) groups stimulated with the 9 mer peptide pool (p52, p61, p68, p69 – 0.1, 1.0 and 10 µg/mL) and the 15 mer peptide pool(p38, p51, p56, p59, p65, p67, p70, p71, p88, p91, p92 - 0.1, 1.0 and 10 µg/ml). After 5 days culture, supernatants were harvested and assessed for levels of IFN-γ by ELISA. Each circle indicates an individual and the dash the median.

The use of pools of class I and class II-restricted *M. leprae*-specific peptides is a necessary step towards a more simplified test. For evaluating the responses to the pools, groups expected to display different levels of response to *M.leprae* were tested: healthy controls from the hyperendemic area (Bom Jardim; EC_high_), the medium leprosy detection rate area (Meireles; EC_low_), household contacts of MB patients (HCMB), and PB patients (**Figure 6**). A second aspect evaluated with the use of peptide pools was the use of 3 peptide concentrations, in a 0.1 to 10 µg/mL range. The responses to the class I-restricted peptides (9 mers) were markedly higher and applied to more individuals when the EC_low_ were compared to the EC_high_. The highest level of stimulation was required for inducing IFN-γ responses in some HCMB and PB patients. The class II-restricted stimulation induced responsiveness and peak levels of IFN-γ in all the evaluated groups at lower concentrations than the class I-restricted stimulus. The two pools of peptides induced responsiveness in more individuals and at lower doses in the EC_low_ and EC_high_ than in the HCMB and PB groups. Taken together the IFN-γ responses induced by peptide pools suggest that the threshold for *M. leprae*-specific production of IFN-γ increases with increased exposure to *M. leprae*, providing a potential explanation for the decline in *M. leprae*-specific IFN production observed with increased exposure to *M. leprae* (HCMB) or active disease (PB).

## Discussion

Humans constitute the only known reservoir of *M. leprae*, except in those select areas with zoonotic leprosy in armadillo populations [16]. It is generally assumed that all diseased individuals must have contracted leprosy directly or indirectly from another infected person. However, the inability to recognize subclinical or latent infections in association with the long incubation time have hampered our knowledge about the mode and source of *M. leprae* transmission and the risk factors associated with disease manifestation among infected individuals. In the previous [13] and present study, we showed that a set of *M. leprae* MHC class I and class II-restricted peptides can specifically identify individuals exposed to *M. leprae* infection and with active disease. The set of *M. leprae*-specific peptides clearly differentiated individuals from an endemic area (Fortaleza) from those living in a non-endemic site for leprosy in Brazil, but with endemic tuberculosis (Porto Alegre).

Our previous [13] and present studies were conducted in distinct endemic sites of Brazil (Rio de Janeiro and Fortaleza), suggesting that despite differences in genetic background, a very similar combination of peptides could efficiently discriminate between exposed and unexposed individuals in other endemic countries. We propose as a major application for this test its use as an epidemiological tool by National Leprosy Control Programs, to define the magnitude of the infected population and consequently of transmission in an endemic area for leprosy. Currently a calibration curve of leprosy new case detection rate versus IFN-γ levels is under construction by evaluating sites with increasing annual leprosy new case detection rates. In addition these peptides are also being analyzed in other leprosy endemic areas in Ethiopia and Asia in order to estimate their use on a worldwide basis.

As a positive response to PGL-I, a specific marker of *M. leprae* is an indicator of bacillary load, the combination of these two observations pointed to a role for *M. leprae* or *M. leprae* components in negatively modulating IFN-γ production in infected individuals, and perhaps contributing to the evolution from infection to active disease in *M. leprae*-exposed individuals.

**Figure 7:**
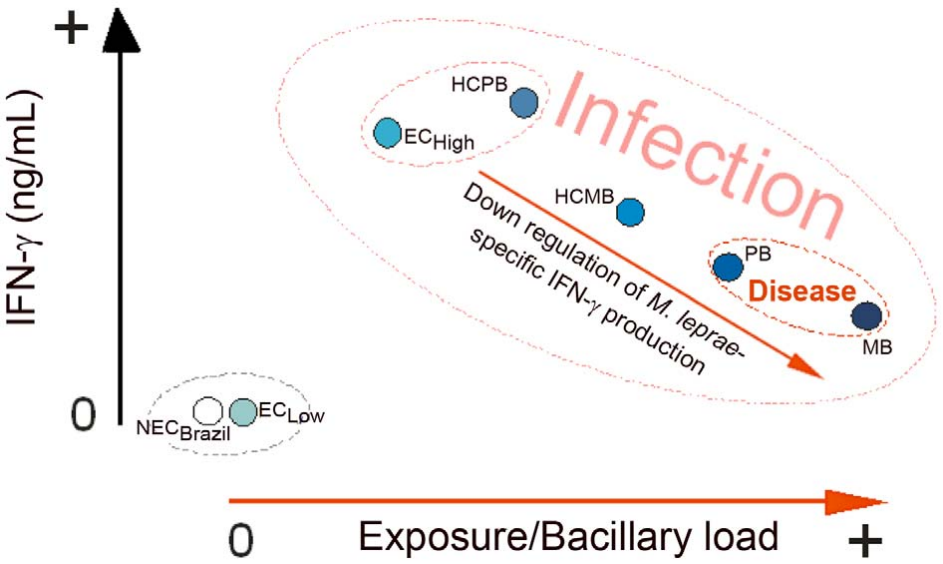
A model for modulation of IFN-γ production during asymptomatic *M. leprae* infection and active disease.

These observations were combined to elaborate a model relating IFN-γ production with the initially asymptomatic *M. leprae* infection, and as the infection progresses to disease, a down regulation of *M. leprae*-specific IFN-γ production (**Figure 7**). The relative IFN-γ levels of the different groups were derived from the median values shown in **Figure 5A**. This observation suggested a new role for the continuous exposure to live *M. leprae* seen in contacts of multibacillary leprosy patients, not only allowing M. *leprae* infection of the HCMB, but also negatively modulating the immune response to this bacillus, available from an exogenous source in the HCMB, or an endogenous source for the PB and MB patients. The distribution of groups in **Figure 7** also raised the possibility that if we want to evaluate immune response in the asymptomatic phase of *M. leprae* infection, patients are not the best option for positive responses. Of note, a similar graph was generated based on the production of IFN-γ in response to the whole bacteria (Figure 4B; except for groups living in sites with low/medium prevalence rates in which a higher background is seen with whole bacteria probably due to cross reactivity), indicating that the immune response to the peptides follows a similar trend as to the whole bacterium.

The proposed model associated the NEC_Brazil_ and EC_low_ groups as the individuals with no or reduced exposure to *M. leprae* infection. A second group can include healthy controls from high endemicity areas (EC_high_) and contacts of PB patients (HCPB). The HCMB group already had intermediate values for IFN-γ, and the patients were at the lower end among the groups of infected individuals. So we tested by ANN the possibility of discriminating infected individuals from patients. The group combining EC_high_ and HCBP was evaluated against MB and PB patients and the decline in IFN-γ levels allowed the correct discrimination of patients in 84.21% of the cases.

A very interesting observation among the groups of non-contact healthy individuals was the correlation between the levels of IFN-γ produced by each group and the degree of exposure to *M. leprae*. While the IFN-γ levels were (almost) absent in individuals living in areas with low/medium prevalence rates (NEC_Brazil_ and EC_low_ groups), in residents of high-prevalence neighborhoods of Fortaleza (EC_high_ group), levels were comparable to those seen in household contacts of leprosy patients. These data indicate that in areas with high prevalence rates, the exposure to *M. leprae* is independent of a previous history of contact with leprosy patients. These results are in agreement with previous studies indicating widespread *M. leprae* nasal carriage as determined by PCR among the general population in an area in which leprosy is endemic [17]. Moreover, they support the view that prolonged intimate contact with a leprosy patient is not required for transmission as has been shown in studies on medical personnel [5], and may explain why a good proportion of incident cases arise among individuals with no previous history of contact with leprosy [18], [19]. Our data also support the general view that *M. leprae* is highly infectious but poorly pathogenic and that most individuals exposed to *M.leprae* present a subclinical infection and develop a protective immune response against this bacillus.

Although close contact is not critical for infection, it seems to play a key role in leprosy manifestation. The critical role of IFN-γ in controlling *M. leprae* infection was first described by Nogueira et al. [20] who demonstrated that lepromatous leprosy and borderline lepromatous patients, in deep contrast to tuberculoid patients, failed to release this cytokine in response to specific antigen. In our study, as shown in Figure 5 and 6, the peak of IFN-γ median production was observed in household contacts of paucibacillary patients (HCPB). Starting from this group, the IFN-γ levels in response to *M. leprae* or *M. leprae*-specific peptides is progressively reduced when groups of increasing levels of exposure to *M. leprae* are compared (HCMB), and are further diminished in leprosy patients. The relatively lower response of contacts of multibacillary patients in comparison to contacts of paucibacillary patients suggests that the evolution of latent infection to active disease is associated with progressive reduction in pathogen-specific IFN-γ production, perhaps in parallel with increase in bacillary load. This down modulation of effector response to *M. leprae* (Ex. IFN-γ levels) in consequence of long-term and constant stimulation of the immune system by the exogenous bacillus released by the index case is a possible explanation for the well-known increased risk of household contacts of multibacillary leprosy patients to develop leprosy [18], [21]. Indeed, the observation that “super exposure” to *M. leprae* can lead to a decrease in host resistance was first described in 1973 [5]. In this study, the authors used a lymphocyte transformation test to show that contacts of lepromatous patients with active disease displayed lower in vitro responses to *M. leprae* when compared with contacts of lepromatous patients treated for more than six months.

Interestingly, HCPB with positive serology to PGL-I produced significantly lower levels of IFN-γ in response to *M. leprae*-specific peptides when compared to PGL-I negative individuals. No similar influence of levels of anti-PGL-I antibody was observed among HCMB, may be because in this case the IFN-γ levels were already down modulated due to the high bacterial exposure. The level of anti-PGL-1 antibody has been considered as a reliable marker of bacterial load in leprosy patients; anti-PGL-1 levels are associated with the disease spectrum and decline upon treatment (for a review see Oskam *et al.* [4], 2003). Moreover, a higher risk of developing leprosy has been found among household contacts seropositive to anti-PGL1 [22]. Thus, PGL-I serology in association with IFN-γ levels in response to the peptides may constitute a robust test for detecting infected individuals with higher bacterial loads and more risk of developing leprosy. The combination of tests for PGL-I specific antibodies and IFN-γ in response to *M. leprae*-specific peptides may require a follow-up study for evaluating patterns of response associated with evolution to active disease or protection. This is currently under investigation at various endemic sites (Geluk *et al.*, for IDEAL consortium).

The studies on the immune response and models of leprosy pathogenesis have been concentrated in active cases that constitute less than 1% of the infected population. Some observations point to the inhibition of dendritic cell maturation and the low frequency of DC s in the lepromatous leprosy lesions as examples of the negative modulation of *M. leprae*-specific immune response in leprosy [23]. But, as seen in our observations in contacts of MB patients and endemic controls of hyperendemic areas, prior to active disease, PBMC from *M. leprae*-exposed individuals respond to *M. leprae*-specific stimuli with high IFN-γ levels. So, at least initially, priming to *M. leprae* and differentiation of a Th1 T cell response takes place. But, failure of DCs in inducing priming and Th1 differentiation of *M. leprae* can be a possible mechanism for lower levels of response in HCMB and patients, especially if components of *M. leprae* such as PGL-I are the culprits [24]. In the course of the chronic stimulation of Th1 and other T cell subsets seen in human and murine diseases such as visceral leishmaniasis, there is induction of IL-10 production by the IFN-γ producing T cell and subsequent down regulation of Th1 differentiation [25], [26]. This is a mechanism that could be relevant for the modulation of response in *M. leprae* latent infection, with potential relevance for the development of a prognostic test and/or vaccines for leprosy.

## Supporting Information

Figure S1 **IFN-γ production in response to PPD or SEB.** The ex vivo stimulation of PBMC was done as described in Fig. 2 legend. Medians of the IFN-γ levels induced by PPD (**A**) or SEB (**B**) are shown for groups displayed in increasing order of exposure to *M. leprae* from left to right in the “x” axis.
(TIF)

Figure S2 **Responsiveness to** ***M. leprae*** **-specific peptides in household contacts of multibacillary leprosy patients (HCMB).** IFN-γ levels in response to the 17 *M. leprae*-specific peptides of PGL-I-positive and PGL-I-negative HCMB are shown in plots **A** and **B**. Detection of IgM anti-PGL1 in sera was performed using a specific ELISA (No significative differences at a p<0.05 level were seen between the PGL-I-positive and negative individuals. Kruskal-Wallys test).
(TIF)

Table S1 *M. leprae* peptides predicted to have high binding scores to HLA molecules.
(DOC)

Table S2 Kruskal-Wallys 2-tailed test, p values.
(DOC)
