# Supplementary figures and images for: Pathogen-Specific Epitopes as Epidemiological Tools for Defining the Magnitude of Mycobacterium leprae Transmission in Areas Endemic for Leprosy
Source: PLoS Negl Trop Dis. 2012 Apr 24;6(4):e1616. doi: 10.1371/journal.pntd.0001616 (PMC3335884; doi:10.1371/journal.pntd.0001616)

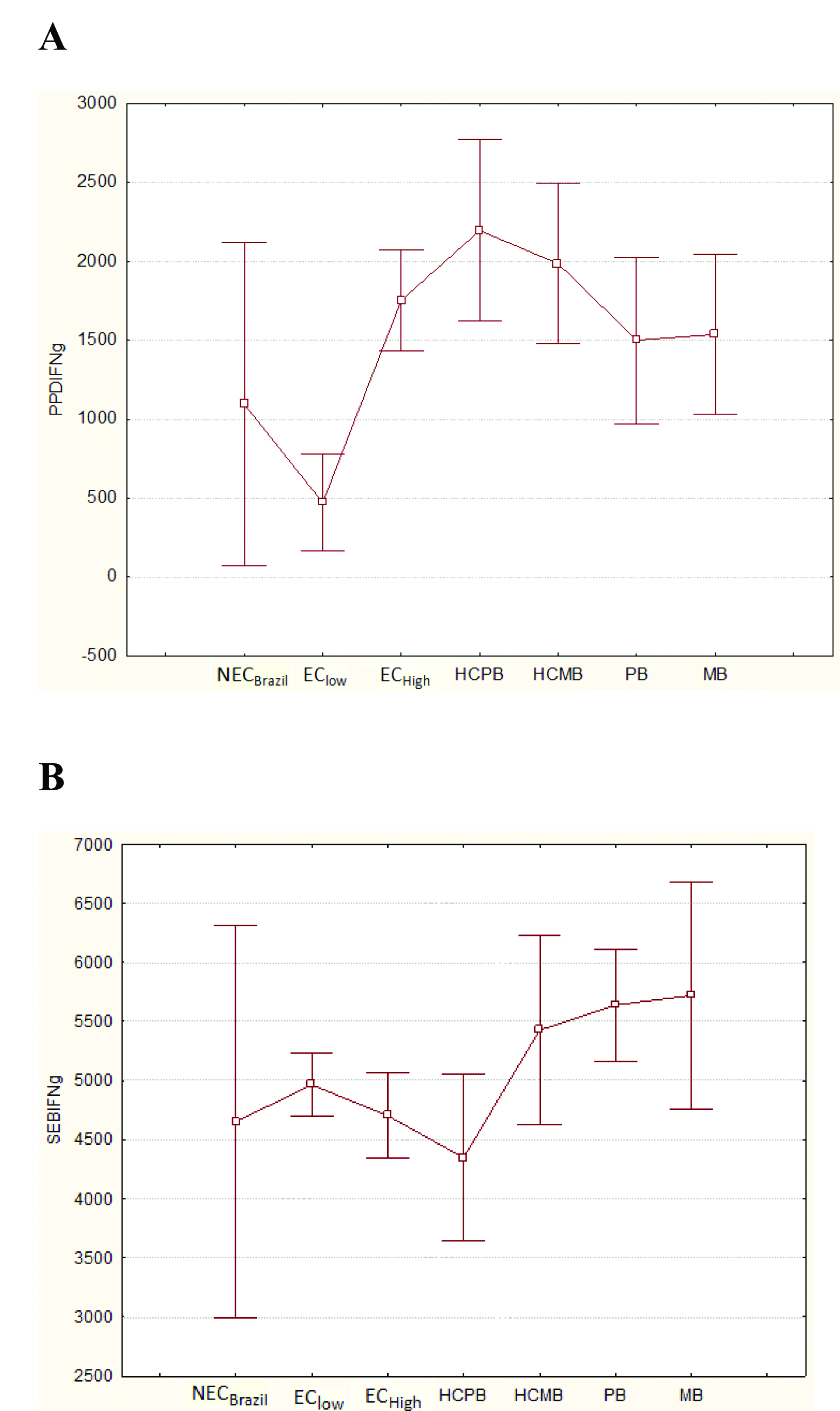

Supplement: Figure S1 — IFN-γ production in response to PPD or SEB. The ex vivo stimulation of PBMC was done as described in Fig. 2 legend. Medians of the IFN-γ levels induced by PPD (A) or SEB (B) are shown for groups displayed in increasing order of exposure to M. leprae from left to right in the “x” axis. (TIF) [file pntd.0001616.s001.tif]

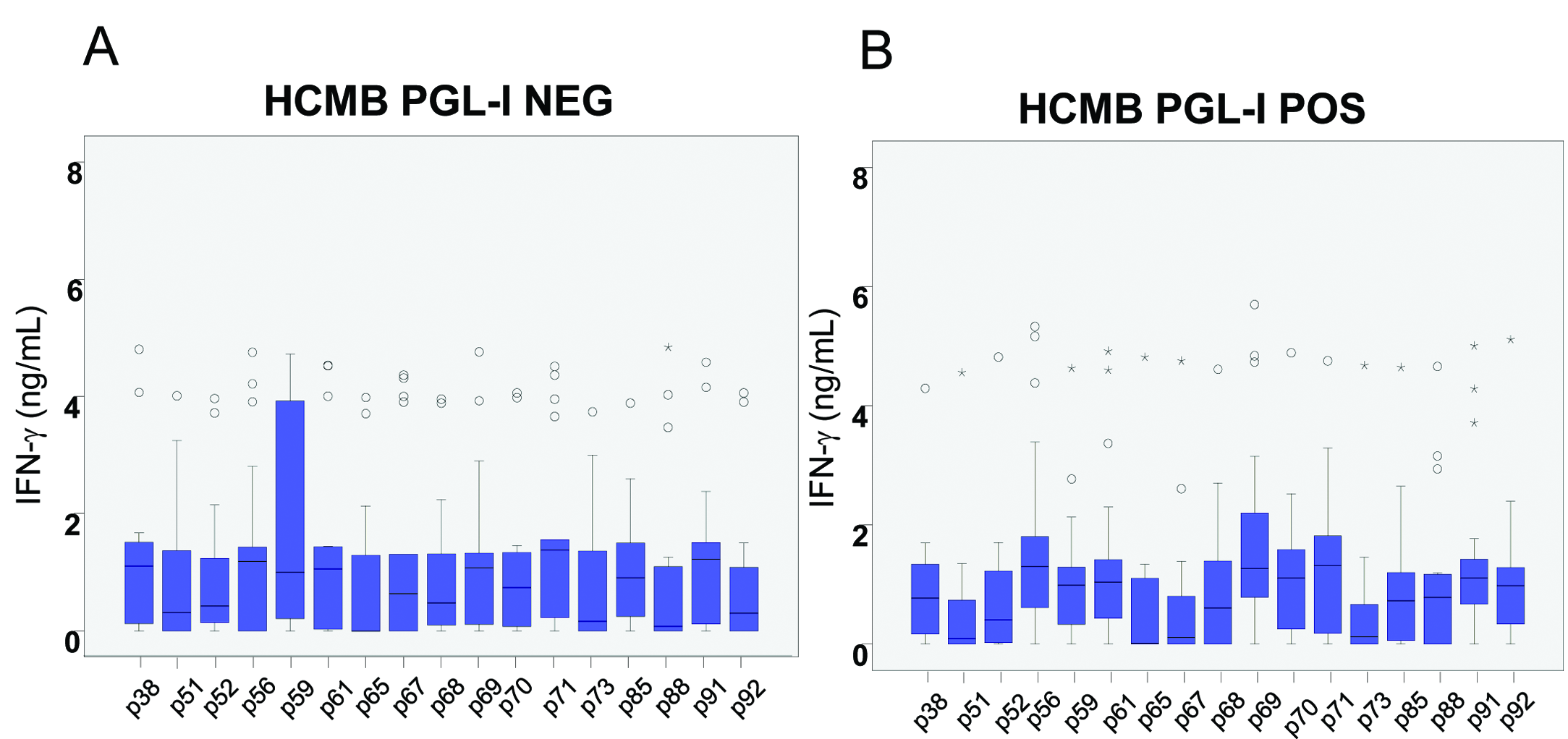

Supplement: Figure S2 — Responsiveness to M. leprae -specific peptides in household contacts of multibacillary leprosy patients (HCMB). IFN-γ levels in response to the 17 M. leprae-specific peptides of PGL-I-positive and PGL-I-negative HCMB are shown in plots A and B. Detection of IgM anti-PGL1 in sera was performed using a specific ELISA (No significative differences at a p<0.05 level were seen between the PGL-I-positive and negative individuals. Kruskal-Wallys test). (TIF) [file pntd.0001616.s002.tif]
