# Supplementary material for: Pathogen-Specific Epitopes as Epidemiological Tools for Defining the Magnitude of Mycobacterium leprae Transmission in Areas Endemic for Leprosy
Source: PLoS Negl Trop Dis. 2012 Apr 24;6(4):e1616. doi: 10.1371/journal.pntd.0001616 (PMC3335884; doi:10.1371/journal.pntd.0001616)
Supplement: Table S1 — M. leprae peptides predicted to have high binding scores to HLA molecules. (DOC) [file pntd.0001616.s003.doc]

| Peptides (designated according to Spencer et al, 2005) | ID/gene | Location | Sequence | Performance ratios (PR)  Rio de Janeiro | Performance ratios (PR)  Fortaleza | Rank  Rio de Janeiro | Rank  Fortaleza |
| --- | --- | --- | --- | --- | --- | --- | --- |
| 37 | ML0008c | aa1-15 | MATIRTVRNLKLCNP |  | 1.00 |  |  |
| 38 | ML0008c | aa21-35 | TRLLTVVVKQRSKAF |  |  |  | 8 |
| 39 | ML0008c | aa24-38 | LTVVVKQRSKAFRPS |  |  |  |  |
| 40 | ML0008c | aa107-121 | RVSYGSECRSGNCLR |  |  |  |  |
| 41 | ML0008c | aa2-10 | ATIRTVRNL |  |  |  |  |
| 42 | ML0126 | aa250-264 | LDDFLSLQRSISPNS |  |  |  |  |
| 43 | ML0126 | aa90-104 | ATHYFEMTSDAFFAD |  |  |  |  |
| 44 | ML0126 | aa251-265 | DDFLSLQRSISPNSY |  |  |  |  |
| 45 | ML0126 | aa65-79 | DVKIAVDPACKLSAR |  |  |  |  |
| 46 | ML0394c | aa117-131 | HLILRITPGIDLREL |  |  |  |  |
| 47 | ML0394c | aa73-87 | LNQLVSLVKQIFVQQ |  |  |  |  |
| 48 | ML0394c | aa22-36 | QLMYLIEITSETKAL |  |  |  |  |
| 49 | ML1057 | aa10-24 | GRYYAEINSAKMYFG |  |  |  |  |
| 50 | ML1057 | aa46-60 | VLVLVGDEAAALEQL |  |  |  |  |
| 51 | ML1057 | aa54-68 | AAALEQLLGQTADVA | 1.91 | 1.01 | 6 | 8 |
| 52 | ML1057 | aa59-67 | QLLGQTADV |  | 0.99 |  | 13 |
| 53 | ML2567 | aa95-109 | RRTVKILRPLPSNIT |  |  |  |  |
| 54 | ML2567 | aa72-86 | NHAVSSDFKTRSTNT |  |  |  |  |
| 55 | ML2567 | aa128-136 | DIAARLASL |  |  |  |  |
| 56 | ML0308 | aa17-31 | FDEYRAMFALSAMDL | 1.51 | 1.00 | 10 | 9 |
| 57 | ML0308 | aa139-153 | THAFDLVLSSHLLFT |  |  |  |  |
| 58 | ML0308 | aa194-208 | DDLLARLRADGVHGE |  |  |  |  |
| 59 | ML0398c | aa15-29 | MLILGLLPAILPACG | 1.64 | 1.06 | 7 | 3 |
| 60 | ML0398c | aa288-302 | AILYRKIHGQDPAER |  |  |  |  |
| 61 | ML0398c | aa16-24 | LILGLLPAI | 1.63 | 1.02 | 8 | 4 |
| 62 | ML0678c | aa31-45 | PYRFHSLAAIWALSP |  |  |  |  |
| 63 | ML0757c | aa6-20 | GINLPKDELTAFGRK |  |  |  |  |
| 64 | ML0757c | aa74-88 | VGAVRIVGGVRPQNF |  |  |  |  |
| 65 | ML1419c | aa108-122 | EAVLLRLDGTTLEVE |  | 1.01 |  | 7 |
| 66 | ML1419c | aa137-151 | QVIFRDLTTQKAAEE |  |  |  |  |
| 67 | ML1419c | aa51-65 | SGRVTYLNPVGVKWM | 1.01 | 1.00 | 12 | 11 |
| 68 | ML1419c | aa267-275 | LLEEGVIVL | 2.69 |  | 2 |  |
| 69 | ML1419c | aa113-121 | RLDGTTLEV | 1.60 |  | 9 |  |
| 70 | ML1420 | aa93-107 | MQEYRGLTSHTPCCR | 3.00 | 0.98 | 1 | 15 |
| 71 | ML1553 *proS* | aa180-194 | LDIYTTLARDMAAIP | 1.09 | 0.99 | 11 | 12 |
| 72 | ML1553 *proS* | aa154-168 | TIEFLWQEGHSAHIE |  |  |  |  |
| 73 | ML1829 | aa108-122 | DAEWLKLTSLGLRPR |  | 1.00 |  | 10 |
| 74 | ML1915 | aa89-104 | VKAVVDDVNAILLTGR |  |  |  |  |
| 75 | ML2177c | aa169-183 | LQPYRLLRGGDSEYW |  |  |  |  |
| 76 | ML2498 | aa75-89 | VGKVQGLLARLLTLP |  |  |  |  |
| 77 | ML2703  *trxB* | aa331-335 | DSTDTTDWSTAMTDA |  |  |  |  |
| 78 | ML0411 | aa264-278 | GLDSIISSASASLLT |  |  |  |  |
| 79 | ML0098 *fbpC* | aa84-98 | GNAMTTLGGRGISVV |  |  |  |  |
| 80 | ML0126 | aa125-139 | YGQVVRDVENTLRYL |  |  |  |  |
| 81 | ML0126 | aa180-194 | GDVWKSIVHLRSTRH |  |  |  |  |
| 82 | ML0394c | aa141-155 | KKRLTLLSGAMARRA |  |  |  |  |
| 83 | ML0394c | aa97-111 | DATFQVIFSQHVHLS |  |  |  |  |
| 84 | ML0638 | aa1-15 | MIDYNNVFGAGVVAA |  |  |  |  |
| 85 | ML0638 | aa49-63 | NYEVSPIFARWPRNR | 2.20 | 1.01 | 4 | 6 |
| 86 | ML0840c | aa287-301 | VYLYNYLLAETSHVL |  |  |  |  |
| 87 | ML0840c | aa251-265 | YRYYRLIATTDAASP |  |  |  |  |
| 88 | ML1189c | aa55-69 | DDIWRTLASAVITGN | 2.19 | 1.18 | 5 | 1 |
| 89 | ML1189c | aa10-24 | FDSFDRILKARSPEA |  |  |  |  |
| 90 | ML2347 | aa20-34 | KENVIIVAAKISWTL |  |  |  |  |
| 91 | ML2347 | aa301-315 | LATVQYDDRRRFTKE | 2.53 | 1.10 | 3 | 2 |
| 92 | ML2452c | aa28-42 | LQAYSNLFGRTSAMQ |  | 0.98 |  | 14 |
| 93 | ML2591 *mce1C* | aa62-76 | GMDVGKVEALKIDGD |  |  |  |  |
| 94 | ML2596 | aa165-179 | WASVALLTAAGVSLG |  |  |  |  |

Table S1. *M. leprae* peptides predicted to have high binding scores to HLA MHC molecules and tested for IFN-γ responses in our previous study( ) were ranked by ANN modeling for their potential to discriminate M.leprae infected/exposed individuals. The information on all of the genes, sequences, and class was obtained from http://www.genolist.pasteur.fr/Leproma/.
