# Supplementary material for: Pathogen-Specific Epitopes as Epidemiological Tools for Defining the Magnitude of Mycobacterium leprae Transmission in Areas Endemic for Leprosy
Source: PLoS Negl Trop Dis. 2012 Apr 24;6(4):e1616. doi: 10.1371/journal.pntd.0001616 (PMC3335884; doi:10.1371/journal.pntd.0001616)
Supplement: Table S2 — Kruskal-Wallys 2-tailed test, p values. (DOC) [file pntd.0001616.s004.doc]

|  | **p38** | | | | | | |
| --- | --- | --- | --- | --- | --- | --- | --- |
|  | MB | PB | HCMB | HCPB | EChigh | EClow | NECBrazil |
| MB |  | 1.000 | 0.247 | **0.001** | 0.061 | 1.000 | 0.656 |
| PB | 1.000 |  | 0.115 | **<0.001** | **0.029** | 1.000 | 1.000 |
| HCMB | 0.247 | 0.115 |  | 1.000 | 1.000 | **<0.001** | **<0.001** |
| HCPB | **0.001** | **<0.001** | 1.000 |  | 1.000 | **<0.001** | **<0.001** |
| EC_high_ | 0.061 | **0.029** | 1.000 | 1.000 |  | **<0.001** | **<0.001** |
| EC_low_ | 1.000 | 1.000 | **<0.001** | **<0.001** | **<0.001** |  | 1.000 |
| NEC_Brazil_ | 0.656 | 1.000 | **<0.001** | **<0.001** | **<0.001** | 1.000 |  |
|  | **p51** | | | | | | |
|  | MB | PB | HCMB | HCPB | EChigh | EClow | NECBrazil |
| MB |  | 1.000 | 1.000 | 0.115 | **0.001** | 1.000 | 0.407 |
| PB | 1.000 |  | 1.000 | 0.861 | **0.013** | 1.000 | 0.051 |
| HCMB | 1.000 | 1.000 |  | 0.723 | **0.006** | 1.000 | **0.006** |
| HCPB | 0.115 | 0.861 | 0.723 |  | 1.000 | **0.019** | **<0.001** |
| EC_high_ | **0.001** | **0.013** | **0.006** | 1.000 |  | **<0.001** | **<0.001** |
| EC_low_ | 1.000 | 1.000 | 1.000 | 0.019 | **<0.001** |  | 1.000 |
| NEC_Brazil_ | 0.407 | 0.051 | **0.006** | **<0.001** | **<0.001** | 1.000 |  |
|  | **p52** | | | | | | |
|  | MB | PB | HCMB | HCPB | EChigh | EClow | NECBrazil |
| MB |  | 1.000 | 0.345 | **0.036** | **0.012** | 1.000 | 1.000 |
| PB | 1.000 |  | 1.000 | 1.000 | 0.759 | 0.170 | **0.050** |
| HCMB | 0.345 | 1.000 |  | 1.000 | 1.000 | **0.003** | **<0.001** |
| HCPB | **0.036** | 1.000 | 1.000 |  | 1.000 | **<0.001** | **<0.001** |
| EC_high_ | **0.012** | 0.759 | 1.000 | 1.000 |  | **<0.001** | **<0.001** |
| EC_low_ | 1.000 | 0.170 | **0.003** | **<0.001** | **<0.001** |  | 1.000 |
| NEC_Brazil_ | 1.000 | **0.050** | **<0.001** | **<0.001** | **<0.001** | 1.000 |  |
|  | **p56** | | | | | | |
|  | MB | PB | HCMB | HCPB | EChigh | EClow | NECBrazil |
| MB |  | 1.000 | 1.000 | 0.985 | 0.327 | 0.054 | 1.000 |
| PB | 1.000 |  | 1.000 | 1.000 | 1.000 | **0.001** | **0.044** |
| HCMB | 1.000 | 1.000 |  | 1.000 | 1.000 | **<0.001** | **0.017** |
| HCPB | 0.985 | 1.000 | 1.000 |  | 1.000 | **<0.001** | **0.002** |
| EC_high_ | 0.327 | 1.000 | 1.000 | 1.000 |  | **<0.001** | **0.001** |
| EC_low_ | 0.054 | **0.001** | **<0.001** | **<0.001** | **<0.001** |  | 1.000 |
| NEC_Brazil_ | 1.000 | **0.044** | **0.017** | **0.002** | **0.001** | 1.000 |  |

|  | **P59** | | | | | | |
| --- | --- | --- | --- | --- | --- | --- | --- |
|  | MB | PB | HCMB | HCPB | EChigh | EClow | NECBrazil |
| MB |  | 1.000 | 0.291 | 0.129 | **0.012** | 0.642 | 0.619 |
| PB | 1.000 |  | 1.000 | 1.000 | 0.436 | **0.022** | **0.018** |
| HCMB | 0.291 | 1.000 |  | 1.000 | 1.000 | **<0.001** | **<0.001** |
| HCPB | 0.129 | 1.000 | 1.000 |  | 1.000 | **<0.001** | **<0.001** |
| EC_high_ | **0.012** | 0.436 | 1.000 | 1.000 |  | **<0.001** | **<0.001** |
| EC_low_ | **0.642** | **0.022** | **<0.001** | **<0.001** | **<0.001** |  | 1.000 |
| NEC_Brazil_ | 0.619 | **0.018** | **<0.001** | **<0.001** | **<0.001** | 1.000 |  |
|  | **p61** | | | | | | |
|  | MB | PB | HCMB | HCPB | EChigh | EClow | NECBrazil |
| MB |  | 0.797 | 0.321 | 0.370 | 0.095 | 1.000 | 0.273 |
| PB | 0.797 |  | 1.000 | 1.000 | 1.000 | **0.004** | **<0.001** |
| HCMB | 0.321 | 1.000 |  | 1.000 | 1.000 | **0.001** | **<0.001** |
| HCPB | 0.370 | 1.000 | 1.000 |  | 1.000 | **0.001** | **<0.001** |
| EC_high_ | 0.095 | 1.000 | 1.000 | 1.000 |  | **<0.001** | **<0.001** |
| EC_low_ | 1.000 | **0.004** | **0.001** | **0.001** | **<0.001** |  | 1.000 |
| NEC_Brazil_ | 0.273 | **<0.001** | **<0.001** | **<0.001** | **<0.001** | 1.000 |  |
|  | **p65** | | | | | | |
|  | MB | PB | HCMB | HCPB | EChigh | EClow | NECBrazil |
| MB |  | 1.000 | 1.000 | 0.349 | 1.000 | 1.000 | 1.000 |
| PB | 1.000 |  | 1.000 | 0.284 | 1.000 | 1.000 | 1.000 |
| HCMB | 1.000 | 1.000 |  | 0.461 | 1.000 | 0.503 | 1.000 |
| HCPB | 0.349 | 0.284 | 0.461 |  | 1.000 | 0.001 | **0.004** |
| EC_high_ | 1.000 | 1.000 | 1.000 | 1.000 |  | 0.056 | 0.154 |
| EC_low_ | 1.000 | 1.000 | 0.503 | **0.001** | 0.056 |  | 1.000 |
| NEC_Brazil_ | 1.000 | 1.000 | 1.000 | **0.004** | 0.154 | 1.000 |  |
|  | **p67** | | | | | | |
|  | MB | PB | HCMB | HCPB | EChigh | EClow | NECBrazil |
| MB |  | 1.000 | 1.000 | 1.000 | **0.026** | 1.000 | 1.000 |
| PB | 1.000 |  | 1.000 | 1.000 | 0.274 | 1.000 | 0.210 |
| HCMB | 1.000 | 1.000 |  | 1.000 | 0.376 | 1.000 | **0.017** |
| HCPB | 1.000 | 1.000 | 1.000 |  | 0.804 | 0.948 | **0.021** |
| EC_high_ | **0.026** | 0.274 | 0.376 | 0.804 |  | **0.004** | **<0.001** |
| EC_low_ | 1.000 | 1.000 | 1.000 | 0.948 | **0.004** |  | 1.000 |
| NEC_Brazil_ | 1.000 | 0.210 | **0.017** | **0.021** | **<0.001** | 1.000 |  |

|  | **p68** | | | | | | |
| --- | --- | --- | --- | --- | --- | --- | --- |
|  | MB | PB | HCMB | HCPB | EChigh | EClow | NECBrazil |
| MB |  | 1.000 | 0.440 | **0.015** | **<0.001** | 1.000 | 1.000 |
| PB | 1.000 |  | 1.000 | 1.000 | 0.121 | 0.156 | **0.037** |
| HCMB | 0.440 | 1.000 |  | 1.000 | 0.102 | **0.022** | **0.003** |
| HCPB | **0.015** | 1.000 | 1.000 |  | 1.000 | <0.001 | <0.001 |
| EC_high_ | **<0.001** | 0.121 | 0.102 | 1.000 |  | <0.001 | <0.001 |
| EC_low_ | 1.000 | 0.156 | **0.022** | **<0.001** | **<0.001** |  | 1.000 |
| NEC_Brazil_ | 1.000 | **0.037** | **0.003** | **<0.001** | **<0.001** | 1.000 |  |
|  | **p69** | | | | | | |
|  | MB | PB | HCMB | HCPB | EChigh | EClow | NECBrazil |
| MB |  | 0.483 | 1.000 | 1.000 | 1.000 | **0.013** | **0.028** |
| PB | 0.483 |  | 1.000 | 1.000 | 1.000 | **<0.001** | **<0.001** |
| HCMB | 1.000 | 1.000 |  | 1.000 | 1.000 | **<0.001** | **<0.001** |
| HCPB | 1.000 | 1.000 | 1.000 |  | 1.000 | **<0.001** | **<0.001** |
| EC_high_ | 1.000 | 1.000 | 1.000 | 1.000 |  | **<0.001** | **0.001** |
| EC_low_ | **0.013** | **<0.001** | **<0.001** | **<0.001** | **<0.001** |  | 1.000 |
| NEC_Brazil_ | **0.028** | **<0.001** | **<0.001** | **<0.001** | **0.001** | 1.000 |  |
|  | **p70** | | | | | | |
|  | MB | PB | HCMB | HCPB | EChigh | EClow | NECBrazil |
| MB |  | 1.000 | 1.000 | 0.270 | **0.037** | 0.401 | 0.405 |
| PB | 1.000 |  | 1.000 | 1.000 | 1.000 | **0.002** | **0.001** |
| HCMB | 1.000 | 1.000 |  | 1.000 | 1.000 | **0.001** | **<0.001** |
| HCPB | 0.270 | 1.000 | 1.000 |  | 1.000 | **<0.001** | **<0.001** |
| EC_high_ | **0.037** | 1.000 | 1.000 | 1.000 |  | **<0.001** | **<0.001** |
| EC_low_ | 0.401 | **0.002** | **0.001** | **<0.001** | **<0.001** |  | 1.000 |
| NEC_Brazil_ | 0.405 | **0.001** | **<0.001** | **<0.001** | **<0.001** | 1.000 |  |
|  | **p71** | | | | | | |
|  | MB | PB | HCMB | HCPB | EChigh | EClow | NECBrazil |
| MB |  | 1.000 | 1.000 | 0.552 | 0.540 | 0.086 | **0.017** |
| PB | 1.000 |  | 1.000 | 1.000 | 1.000 | **0.012** | **0.002** |
| HCMB | 1.000 | 1.000 |  | 1.000 | 1.000 | **<0.001** | **<0.001** |
| HCPB | 0.552 | 1.000 | 1.000 |  | 1.000 | **<0.001** | **<0.001** |
| EC_high_ | 0.540 | 1.000 | 1.000 | 1.000 |  | **<0.001** | **<0.001** |
| EC_low_ | 0.086 | **0.012** | **<0.001** | **<0.001** | **<0.001** |  | 1.000 |
| NEC_Brazil_ | **0.017** | **0.002** | **<0.001** | **<0.001** | **<0.001** | 1.000 |  |

|  | **p73** | | | | | | |
| --- | --- | --- | --- | --- | --- | --- | --- |
|  | MB | PB | HCMB | HCPB | EChigh | EClow | NECBrazil |
| MB |  | 1.000 | 0.972 | 0.171 | **<0.001** | 1.000 | 1.000 |
| PB | 1.000 |  | 1.000 | 1.000 | **0.023** | 1.000 | 0.217 |
| HCMB | 0.972 | 1.000 |  | 1.000 | **0.024** | 1.000 | **0.018** |
| HCPB | 0.171 | 1.000 | 1.000 |  | 0.461 | 0.446 | **0.002** |
| EC_high_ | **<0.001** | **0.023** | **0.024** | 0.461 |  | **<0.001** | **<0.001** |
| EC_low_ | 1.000 | 1.000 | 1.000 | 0.446 | **<0.001** |  | 1.000 |
| NEC_Brazil_ | 1.000 | 0.217 | **0.018** | **0.002** | **<0.001** | 1.000 |  |
|  | **p85** | | | | | | |
|  | MB | PB | HCMB | HCPB | EChigh | EClow | NECBrazil |
| MB |  | 0.734 | **0.006** | **0.012** | 0.515 | 1.000 | 1.000 |
| PB | 0.734 |  | 1.000 | 1.000 | 1.000 | 1.000 | **0.025** |
| HCMB | **0.006** | 1.000 |  | 1.000 | 1.000 | 0.446 | **<0.001** |
| HCPB | **0.012** | 1.000 | 1.000 |  | 1.000 | 0.588 | **<0.001** |
| EC_high_ | 0.515 | 1.000 | 1.000 | 1.000 |  | 1.000 | **0.016** |
| EC_low_ | 1.000 | 1.000 | 0.446 | 0.588 | 1.000 |  | 0.713 |
| NEC_Brazil_ | 1.000 | **0.025** | <0.001 | <0.001 | 0.016 | 0.713 |  |
|  | **p88** | | | | | | |
|  | MB | PB | HCMB | HCPB | EChigh | EClow | NECBrazil |
| MB |  | 1.000 | 1.000 | **0.015** | 0.075 | 1.000 | 0.388 |
| PB | 1.000 |  | 1.000 | 0.159 | 0.509 | 1.000 | **0.053** |
| HCMB | 1.000 | 1.000 |  | 0.243 | 0.868 | 1.000 | **0.002** |
| HCPB | **0.015** | 0.159 | 0.243 |  | 1.000 | 0.102 | **<0.001** |
| EC_high_ | 0.075 | 0.509 | 0.868 | 1.000 |  | 0.332 | **<0.001** |
| EC_low_ | 1.000 | 1.000 | 1.000 | 0.102 | 0.332 |  | 0.166 |
| NEC_Brazil_ | 0.388 | **0.053** | **0.002** | **<0.001** | **<0.001** | 0.166 |  |
|  | **p91** | | | | | | |
|  | MB | PB | HCMB | HCPB | EChigh | EClow | NECBrazil |
| MB |  | 1.000 | 1.000 | **0.022** | 1.000 | 1.000 | **0.031** |
| PB | 1.000 |  | 1.000 | 0.873 | 1.000 | 0.374 | **<0.001** |
| HCMB | 1.000 | 1.000 |  | 0.734 | 1.000 | 0.091 | **<0.001** |
| HCPB | **0.022** | 0.873 | 0.734 |  | 1.000 | **<0.001** | **<0.001** |
| EC_high_ | 1.000 | 1.000 | 1.000 | 1.000 |  | **0.035** | **<0.001** |
| EC_low_ | 1.000 | 0.374 | 0.091 | **<0.001** | **0.035** |  | 1.000 |
| NEC_Brazil_ | **0.031** | **<0.001** | **<0.001** | **<0.001** | **<0.001** | 1.000 |  |

|  | **p92** | | | | | | |
| --- | --- | --- | --- | --- | --- | --- | --- |
|  | MB | PB | HCMB | HCPB | EChigh | EClow | NECBrazil |
| MB |  | 1.000 | 1.000 | 0.116 | **0.015** | 1.000 | 1.000 |
| PB | 1.000 |  | 1.000 | 1.000 | 0.555 | 1.000 | 0.084 |
| HCMB | 1.000 | 1.000 |  | 1.000 | 0.741 | 0.496 | **0.005** |
| HCPB | 0.116 | 1.000 | 1.000 |  | 1.000 | **0.026** | **<0.001** |
| EC_high_ | **0.015** | 0.555 | 0.741 | 1.000 |  | **0.003** | **<0.001** |
| EC_low_ | 1.000 | 1.000 | 0.496 | **0.026** | **0.003** |  | 1.000 |
| NEC_Brazil_ | 1.000 | 0.084 | **0.005** | **<0.001** | **<0.001** | 1.000 |  |
|  | ***M. leprae*** | | | | | | |
|  | MB | PB | HCMB | HCPB | EChigh | EClow | NECBrazil |
| MB |  | 0.082 | **0.001** | **<0.001** | **<0.001** | 0.891 | 1.000 |
| PB | 0.082 |  | 1.000 | 1.000 | 1.000 | 1.000 | **0.003** |
| HCMB | **0.001** | 1.000 |  | 1.000 | 1.000 | 1.000 | **<0.001** |
| HCPB | **<0.001** | 1.000 | 1.000 |  | 1.000 | 0.225 | **<0.001** |
| EC_high_ | **<0.001** | 1.000 | 1.000 | 1.000 |  | 0.656 | **<0.001** |
| EC_low_ | 0.891 | 1.000 | 1.000 | 0.225 | 0.656 |  | 0.080 |
| NEC_Brazil_ | 1.000 | **0.003** | **<0.001** | **<0.001** | **<0.001** | 0.080 |  |
|  | **PPD** | | | | | | |
|  | MB | PB | HCMB | HCPB | EChigh | EClow | NECBrazil |
| MB |  | 1.000 | 1.000 | 1.000 | 1.000 | **0.028** | 0.308 |
| PB | 1.000 |  | 1.000 | 1.000 | 1.000 | **0.048** | 0.487 |
| HCMB | 1.000 | 1.000 |  | 1.000 | 1.000 | **<0.001** | **0.009** |
| HCPB | 1.000 | 1.000 | 1.000 |  | 1.000 | **<0.001** | **0.001** |
| EC_high_ | 1.000 | 1.000 | 1.000 | 1.000 |  | **0.001** | **0.016** |
| EC_low_ | **0.028** | **0.048** | **<0.001** | **<0.001** | **0.001** |  | 1.000 |
| NEC_Brazil_ | 0.308 | 0.487 | **0.009** | **0.001** | **0.016** | 1.000 |  |
|  | **SEB** | | | | | | |
|  | MB | PB | HCMB | HCPB | EChigh | EClow | NECBrazil |
| MB |  | 1.000 | 1.000 | 0.393 | 1.000 | 1.000 | 0.176 |
| PB | 1.000 |  | 1.000 | 0.084 | 0.425 | 1.000 | **0.036** |
| HCMB | 1.000 | 1.000 |  | 1.000 | 1.000 | 1.000 | 1.000 |
| HCPB | 0.393 | 0.084 | 1.000 |  | 1.000 | 1.000 | 1.000 |
| EC_high_ | 1.000 | 0.425 | 1.000 | 1.000 |  | 1.000 | 1.000 |
| EC_low_ | 1.000 | 1.000 | 1.000 | 1.000 | 1.000 |  | 1.000 |
| NEC_Brazil_ | 0.176 | **0.036** | 1.000 | 1.000 | 1.000 | 1.000 |  |

Table S2- Kruskal-Wallys 2-tailed test, p values. Differences in IFN-γ levels between groups with p ≤ 0.05 values were deemed significant (Shown in bold). The p values for the comparison between 2 given groups are shown in the intersection between the line and the column displaying the group identifications. The peptides and other stimuli used in the cultures are shown in bold.
